# Rapidly discriminate commercial medicinal *Pulsatilla chinensis* (Bge.) Regel from its adulterants using ITS2 barcoding and specific PCR-RFLP assay

**DOI:** 10.1038/srep40000

**Published:** 2017-01-06

**Authors:** Yuhua Shi, Mingming Zhao, Hui Yao, Pei Yang, Tianyi Xin, Bin Li, Wei Sun, Shilin Chen

**Affiliations:** 1 Key Laboratory of Beijing for Identification and Safety Evaluation of Chinese Medicine, Institute of Chinese Materia Medica, China Academy of Chinese Medical Sciences, Beijing 100700, China; 2 Institute of Medicinal Plant Development, Chinese Academy of Medical Sciences & Peking Union Medical College, Beijing 100193, China; 3 Xi’an Botanical Garden of Shanxi Province, Xi’an 710061, China

## Abstract

Pulsatillae radix is a conventional traditional Chinese medicine (TCM) with common name Baitouweng, and has notable effects on inflammation and dysentery. *Pulsatilla chinensis* (Bge.) Regel is the only source plant of Baitouweng recorded in Chinese Pharmacopoeia, but its adulteration often occurs in the market that possibly affects medicinal efficacy and safety. We have established an internal transcribed spacer 2 (ITS2) barcode library based on 105 plant samples from 12 *Pulsatilla* species and 10 common adulterants. Results indicate that ITS2 barcoding can accurately distinguish *Pulsatilla* species from their adulterants. *Pulsatilla chinensis* can be discriminated from 11 congeneric species by two stable single nucleotide polymorphisms (SNPs) in the ITS2 region. Additionally, a quick specific PCR-RFLP identification assay based on the ITS2 barcode was developed. Using specific primers ITS2/PR1 combined with restriction enzyme *Bgl* I, *Pu. chinensis* can rapidly be differentiated from other species via simple and low-cost test procedures. Furthermore, 30 commercial Baitouweng products were tested and only two products were derived from authentic *Pu. chinensis*. Thus, these two molecular approaches provide practical tools for quick identification of commercial Baitouweng products and can help ensure the safe use of this TCM product.

In the last decade, traditional medicine has had a global expansion and is no longer viewed as merely a health care in developing countries. The World Health Organization estimates that 70–80% of the population in developed countries has used complementary and alternative medicine to manage their health^1^. The huge increase in demand for herbal remedies in recent years demonstrates that herbal medicines are perceived to be as natural and safe. However, fraudulent labeling, counterfeit production, substitution, and adulteration of traditional medicines are becoming increasingly severe, leading to global concern that these activities threaten consumer safety in the absence of more stringent and effective regulation for quality control. TCM herbal materials procured from markets are always in the form of dried or powdered plant parts; therefore, their accurate identification by conventional taxonomy are difficult. Many DNA based methods have been developed for identifying species, which are not dependent on morphological identification by plant specialists^2^. RAPD (random amplified polymorphic DNA), SSR (simple sequence repeat), ISSR (Inter-SSR), AFLP (amplified fragment length polymorphisms) and RFLP (restriction fragment length polymorphism) were the early molecular marker identification methods that have been widely used for identifying species^3^,^4^. However, these methods involved time intensive, and expensive laboratory procedures, making them unsuitable for rapid species identification. DNA barcoding is a technology that uses short and standardized DNA sequences to identify species^5^,^6^. In 2003, the mitochondrial gene cytochrome c oxidase subunit 1 (*COI*), was proposed as a DNA barcode marker for the discrimination of animal species^7^,^8^. Since then, DNA barcoding has aroused great attention and research interest for practical applications^9^,^10^,^11^. The Plant Working Group in botany, the consortium for the barcodes of life (CBOL), recommended that the plastid DNA regions *matK* and *rbcL* act as the core barcodes for land plants^12^, to be supplemented with the plastid *psbA*-*trnH* and the nuclear ribosomal internal transcribed spacer (ITS)^13^. Chen *et al*. reported that the second internal transcribed spacer (ITS2) of the nuclear ribosomal DNA had better species discriminating power and suggested it as the standard DNA barcode to identify medicinal plants^14^,^15^. DNA barcoding can achieve rapid, accurate identification of species from raw materials and has become a reliable molecular tool for authenticating medicinal plants and their adulterants^2^,^4^,^16^,^17^. Meanwhile, several DNA barcoding databases were established and published online, such as the Barcode of Life Data System (http://www.boldsystems.org)^6^, DNA barcoding system for herbal materials (http://www.tcmbarcode.cn/en)^2^, and organism-specific databases for Lepidoptera (http://www.lepbarcoding.org/) and fish (http://www.fishbol.org/). These barcoding systems provide open barcode databases and standard protocols for species identification. In recent years, a lot of quick identification methods based on DNA barcode markers were developed, such as Allele-specific PCR^18^, Real-time PCR^19^, High Resolution Melting (HRM) analysis^20^, PCR-RFLP^21^, and etc. These methods have simpler test procedures and shorter time-consuming than conventional methods that were more convenient for rapid identification.

Pulsatillae radix, Baitouweng (Chinese vernacular name), is well known as a commonly used traditional Chinese medicines (TCM) because of its notable medicinal uses in heat-clearing, blood cooling and detoxification^22^. Recent, chemical studies have found that Baitouweng contains anemonin, triterpenoid saponins, pulsatillic acids, flavonoids, daucosterols, and etc.^23^,^24^,^25^,^26^. Pharmacological research indicated that it had anti-amoebic, antibacterial^27^, and antitumor effects^28^,^29^. However, Baitouweng is an easily confused TCM and its adulteration often occurs in the markets where it is sold to consumers. *Pulsatilla chinensis* (Bge.) Regel of the Ranunculaceae family is the only plant source of Pulsatillae radix recorded officially in the Chinese Pharmacopoeia^22^. In the Flora of China, *Pulsatilla* is regarded as a separate genus differ from *Anemone* because of morphology on the achenes^30^. But molecular phylogenetic studies have shown that *Pulsatilla* is nested within *Anemone*^31^, and *Pulsatilla* is treated as a clade of *Anemone*, e.g. the TROPICOS (www.tropicos.org/Name/27104112) and the Flora of North American (http://www.efloras.org/florataxon.aspx?flora_id=1&taxon_id=101733). Thus, *Pu. chinensis* and *Anemone chinensis* actually represent the same species. Here, we continue to follow use of the name *Pu. chinensis* out of deference to current usage in TCM. There are about 33 *Pulsatilla* species in the world, mainly distributed in Asia, Europe and North America, and 11 *Pulsatilla* species are widely distributed in China throughout the provinces of Yunnan, Sichuan, Qinghai, Shaanxi, Henan, Heilongjiang and Nei Mongol^30^. Besides *Pu. chinensis, Pu. cernua* (Thunb.) Bercht. et Opiz., *Pu. turczaninovii* Kryl. et Serg., *Pu. ambigua* Turcz. ex Pritz., *Pu. dahurica* (Fisch. ex DC.) Spreng., and *Pu. campanella* Fisch. ex Regel have also been referenced as the plant source of medicinal Baitouweng in some Chinese herbal books^32^. In addition, vegetative morphology similar to that of *Pu. chinensis* is found not only in some closely related species (e.g. *Anemone hupehensis* Lem.), but also in considerably more distantly related ones like *Potentilla chinensis* Ser. (recorded as “Huangzhou Baitouweng”) or *Po.discolor* Bge. (recorded as “Lanling Baitouweng”), both of which are in the Rosaceae^33^. Similarly, due to similar appearances of medicinal parts, dry roots from *Rhaponticum uniflorum* (L.) DC. (Compositae) and *Platycodon grandiflorus* (Jacq.) A. DC. (Campanulaceae) have also been mistaken for, and sold as Baitouweng. Theses misuse practices of Baitouweng will not only decrease the pharmacological efficacy but also compromise the medicinal safety, which must be avoided as much as possible. Baitouweng products in the market are in form of pieces or powders and can not be identified by plant morphological characteristics. So far, the main methods for distinguishing them are based on microscopic characteristics^34^,^35^ and physicochemical analysis^36^,^37^, but still difficult to determine specific species. There have been a few molecular studies focused on the phylogenetic relationships of Ranunculaceae based on DNA markers^31^,^38^. Sun *et al*. used the ITS region to distinguish a new *Pulsatilla* species, *Pu. tongkangensis*, from other *Pulsatilla* species^39^. However, research on distinguishing medicinal *Pu. chinensis* from its adulterants is still limited. Therefore, an accurate and convenient identification tool to distinguish authentic medicinal *Pu. chinensis* (Baitouweng) from its adulterants is absolutely essential.

The aim of this study was to explore effective and quick approaches to discriminate the genuine origin of medicinal Baitouweng, *Pu. chinensis*, from its adulterants based on the ITS2 region, and then use these tools to authenticate commercial medicinal Baitouweng products for ensuring the quality of raw materials in the market.

## Results and Discussion

### The ITS2 library of *Pu. chinensis* and its adulterants

Establishment of a DNA barcode database is the precursor for molecular identification of species. To extend the existing ITS2 database, 84 fresh plant samples representing 12 *Pulsatilla* species and 10 adulterant species were collected from different localities in China (see Supplementary Table S1). All these species were identified by taxonomic experts according to the Flora of China (http://foc.eflora.cn/) and deposited in the herbarium of the Institute of Medicinal Plant Development, Chinese Academy of Medical Sciences (IMD). PCR results showed that the ITS2 region of all 84 samples were successfully amplified by universal primers ITS2F/ITS3R and sequenced well. By removing the flanking 5.8S and 28S rRNA gene sequences, 84 ITS2 sequences were obtained and deposited in GenBank under accession numbers KR611721–86, KT969417-31, FJ980329, FJ980329, and FJ980346. To include more genotypes of the sampled species, 21 ITS2 sequences were downloaded from GenBank (see Supplementary Table S1). Because some downloaded sequences were partial, seven nucleotides at the 3′ terminus after sequence alignment were cut off to keep all sequences consistent. Thus, the ITS2 barcode library of *Pu. chinensis* and its common adulterants was successfully established, containing 105 reference ITS2 sequences from 12 *Pulsatilla* species and 10 commonly confused species. Sequence analysis showed that the length of the ITS2 sequences ranged from 204 bp to 255 bp, the average GC contents ranged from 49.8% to 67.0%, the number of variable sites ranged from 0 to 20 (Table 1), and the average intraspecific K2P distance of ITS2 in each species varied from 0 to 0.046 (see Supplementary Table S2). This established library expanded the ITS2 barcodes of *Pu. chinensis* and its common adulterants in our existing DNA barcoding database for herbal materials^2^ and was necessary for identifying the origin species of medicinal Baitouweng.

### Discrimination of *Pu. chinensis* and its adulterants using ITS2 barcoding

ITS2 sequences from 25 individuals of *Pu. chinensis* were obtained in this study. Sequence analysis showed that 25 ITS2 sequences had the same length of 211 bp and the average GC content was 64.5% (Table 1). There were four variable sites (17 bp, 63 bp, 125 bp, and 169 bp) in the ITS2 sequences that segregated as five sequence haplotypes (H): H1 (B1-B13, B15-16), H2 (B20, B22-25), H3 (B17-19), H4 (B14) and H5 (B21) (see Supplementary Fig. S1). Among them, H1 contains 15 sequences, indicating it may be the major ITS2 haplotype of *Pu. chinensis*. Moreover, the Kimura 2-parameter (K2P) intraspecific distances among them had a mean of 0.006, ranging from 0 to 0.019 (Table 2).

**Figure 1:**
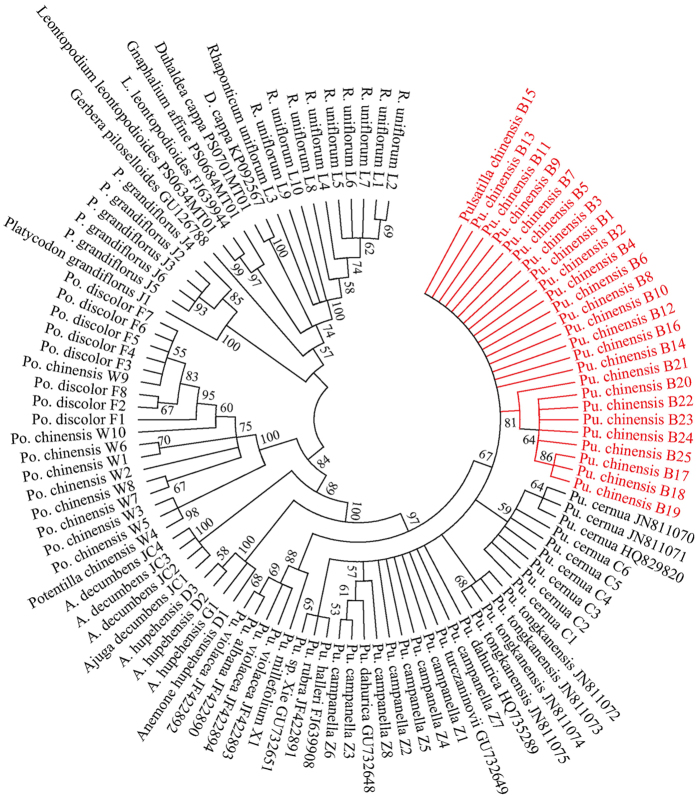
Phylogenetic NJ tree of *Pulsatilla chinensis* and its adulterants constructed with the ITS2 sequences. The bootstrap scores (1000 replicates) are shown (≥50%) for each branch. All voucher numbers are from sample codes in this study and GenBank accession numbers.

Nearest genetic distance, BLAST and neighbor-joining (NJ) tree methods were used to evaluate the identification capability of the ITS2 barcode^15^,^40^. Firstly, we estimated discrimination power of the ITS2 barcode between *Pu. chinensis* and 10 adulterants from other genera. Sequence divergences showed that the K2P interspecific distances between *Pu. chinensis* and these species ranged from 0.113 to 0.755 (Table 2). Thus, the minimum interspecific distance 0.113 was much more than the maximum intraspecific distance of *Pu. chinensis* (0.019), indicating that *Pu. chinensis* can easily be distinguished from these 10 adulterants based on the ITS2 sequences by K2P distance method. NJ tree result also showed that *Pu. chinensis* clustered into one clade with congeneric species and can discriminate from other included taxa (Fig. 1). Moreover, our results also exhibited that *Anemone hupehensis* clustered into one clade with a bootstrap value of 100, and had the closest relationship to *Pulsatilla* species (Fig. 1), and the interspecific distance between them was 0.104. *R. uniflorum, P. grandiflorum, Ajuga decumbens, Leontopodium leontopodioides, Duhaldea cappa, Gerbera piloselloides*, and *Gnaphalium affine* also represented monophyletic groups, but *Po. chinensis* and *Po. discolor* clustered into the same clade with a bootstrap value of 100 (Fig. 1), implying that the ITS2 sequences in these two species were very similar. However, these 10 known adulterants of medicinal Baitouweng can be easily and accurately be discriminated from *Pu. chinensis* using the ITS2 barcode.

**Figure 2:**
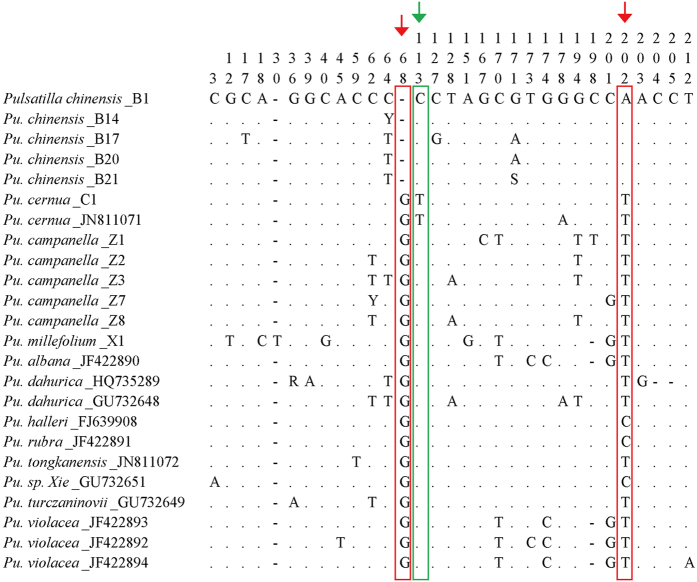
SNPs analysis of *Pulsatilla* species in the ITS2 sequences. Samples represent different ITS2 haplotypes of each *Pulsatilla* species. The number represents the position of variable sites (bp); dots (.) represents the nucleotide identical to the first line; dash (−) represents a nucleotide indel; Y represents T/C; S represents C/G; R represents A/G. Red boxes and arrows indicate SNPs in *Pulsatilla chinensis*, and green boxes and arrow indicate SNPs in *Pu. cernua.*

Secondly, we analyzed identification ability of the ITS2 barcode between *Pu. chinensis* and 11 congeneric species. The ITS2 length 12 *Pulsatilla* species ranged from 210 bp to 212 bp and the average GC content was 64.6% (Table 1). The K2P distance analysis indicated that the minimum interspecific distance between *Pu. chinensis* and 11 congeneric species (0.005) was less than the maximum intraspecific distance of *Pu. chinensis* (0.019) (Table 2). The NJ tree showed that 12 *Pulsatilla* species were clustered into one clade with a bootstrap value of 97 (Fig. 1). These results demonstrated that the 12 *Pulsatilla* species shared similar ITS2 sequences and neither K2P genetic distance nor NJ tree analysis could distinguish them well. Searching the chromosome numbers tabulated in The Chromosome Counts Database (CCDB)^41^ showed that most of the examined *Pulsatilla* species were diploids (n = 8), that probably why the ITS2 sequences shows little variation. Furthermore, by comparing different ITS2 haplotypes from the 12 *Pulsatilla* species, 32 single nucleotide polymorphism (SNP) sites were found in them, and two SNP sites were distinctive in five ITS2 haplotypes of *Pu. chinensis*, which were a G nucleotide deletion at position 68 and an A nucleotide variation at position 202 (Fig. 2). Thus, although there was a high similarity among ITS2 sequences of *Pulsatilla* species, the two specific and stable SNP sites can still effectively discriminate *Pu. chinensis* from the sampled congeneric species. Meanwhile, SNP analysis of the ITS2 region detected one specific SNP site at position 113 with a T nucleotide variation in *Pu. cernua* (Fig. 2), which provides a fast molecular identification method for that species.

In summary, ITS2 barcoding can distinguish *Pu. chinensis* from its known adulterants. However, there are still challenges for distinguishing closely related species based on DNA barcoding^42^. In this study, some *Pulsatilla* species and two *Potentilla* species (*Po. chinensis* and *Po. discolor*) could not be discriminated well by the ITS2 barcode (Fig. 1). The poor resolution of ITS2 and other barcodes were also reported in the genus *Crataegus*^43^. Thus, more work is necessary to expand the reference plant data set, and screening of more supplementary DNA loci to identify specific barcodes for difficult genera.

### Authentication of commercial Baitouweng products through ITS2 barcoding

**Figure 3:**
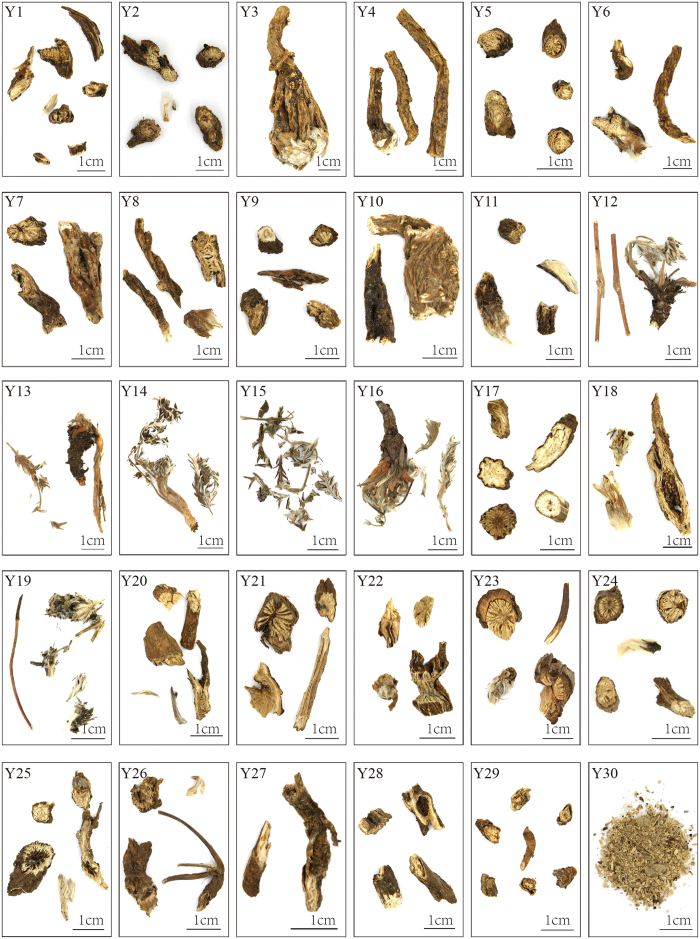
Photos of commercial Baitouweng products. Y1 to Y30 represent sample codes of commercial products. Scale bar, 1 cm.

A total of 30 Baitouweng products in the market were surveyed in this study to confirm the identification efficiency of ITS2 barcoding. These products were purchased in medicinal herb markets and stores in the cities of Baoding, Shijiazhuang, Nanning, Beijing, Anguo, Yulin, and Bozhou in China and numbered as Y1 to Y30. They were in the forms of dry root pieces, leaves, or powder, with no morphologically recognizable features that could allow species identification (Fig. 3). Our results showed that ITS2 sequences of 30 commercial products were all successfully amplified by the universal primers ITS2F/3R. Among them, ITS2 sequences of Y1 to Y29 were obtained by directly sequencing the purified PCR products. Direct sequencing of Y30 failed because of double sequence peaks, implying that Y30 might be a mixture containing several taxa. Eventually, 17 ITS2 sequences were obtained from Y30 by using the TA cloning method. Sequence alignments showed that these sequences divided into two types, nine sequences were 216 bp in length and the other eight ones were 210 bp in length. Therefore, all ITS2 sequences from 30 commercial products were finally obtained. ITS2 length ranged from 211 bp to 255 bp (Table 3), suggesting that Y1 to Y30 were not just derived from one species.

**Figure 4:**
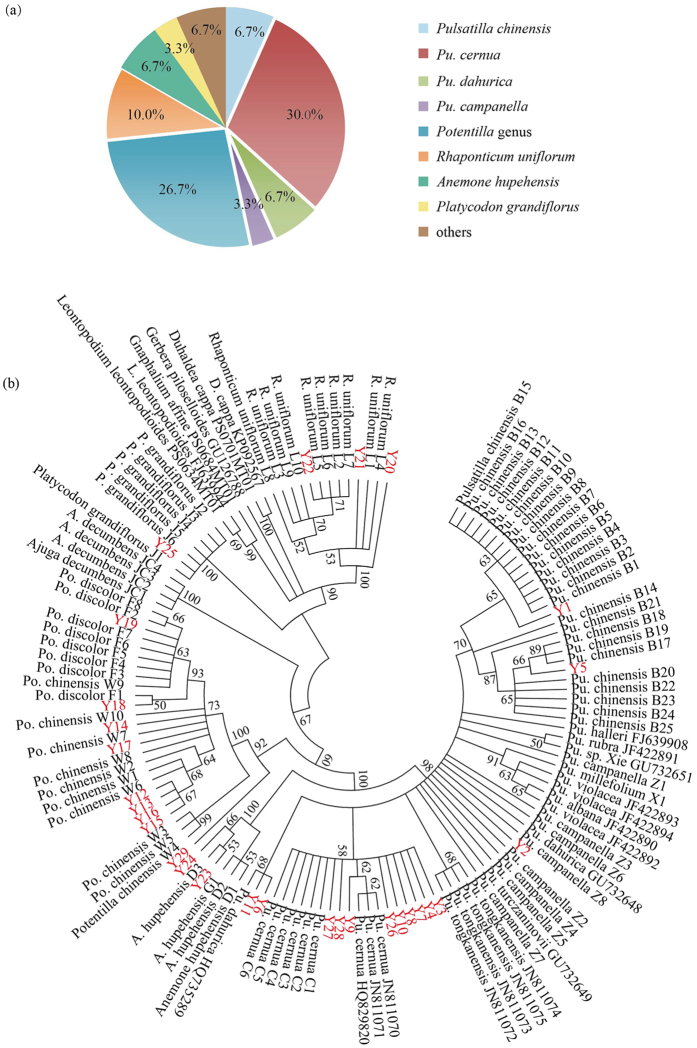
Authentication of commercial Baitouweng products by ITS2 barcoding. **(a**) Species diversity of commercial Baitouweng products. (**b**) Phylogenetic NJ tree of voucher species and commercial products based on the ITS2 sequences. The bootstrap scores (1000 replicates) are shown (≥50%) for each branch. Y1 to Y30 represent sample codes of commercial products, others are sample codes of voucher samples and GenBank accession numbers.

BLAST and NJ tree methods were used to authenticate commercial Baitouweng products. All ITS2 sequences were aligned using the BLAST algorithm to search for the highest sequence similarity amongst samples to identify the species based on the established ITS2 library of *Pu. chinensis* and its adulterants, and online databases (http://www.tcmbarcode.cn/en/, and http://blast.ncbi.nlm.nih.gov/Blast.cgi). The results indicated Y1 and Y5 shared 100% and 99.5% homology with *Pu. chinensis*, respectively (Table 3), and had the two identical SNP sites as *Pu. chinensis* (see Supplementary Fig. S2). Thus, Y1 and Y5 can be authenticated as *Pu. chinensis*. Nine products (Y3, Y4, Y7, Y8, Y9, Y10, Y26, Y28 and Y29) shared 100% identity with *Pu. cernua* and contained the same SNP site at position 113 (see Supplementary Fig. S2), thus were identified as *Pu. cernua.* Three products had the most similarity with *Pu. dahurica* (Y6 and Y11) and *Pu. campanella* (Y2) (Table 3). In addition, eight products were likely derived from the genus *Potentilla*, sharing 98.1–100% sequence similarity with *Po. chinensis, Po. nivea*, and *Po. Discolor*. Seven products were likely derived from *R. uniflorum* (3), *A. hupehensis* (2), *P. grandiflorus* (1), and *Gossypium* species (1). For product of Y30, nine sequences shared 98.2% to 100% homology with *R. uniflorum* and the other eight sequences shared 99.1 to 99.5% similarity with *Astragalus* species, indicating that Y30 was mixed with at least two species. In summary, two Baitouweng products (6.7%) were derived from *Pu. chinensis*, 12 from congeneric species, and 16 from other species (Fig. 4a). NJ tree analysis was consistent with the BLAST result (Fig. 4b).

Our market survey revealed that there were a number of adulterants of medicinal Baitouweng in the market, causing serious potential health risks for users. These adulterants were mainly divided into two types. Type one are substitutes from congeneric species. Our study showed 12 Baitouweng products were from congeners of *Pu. chinensis*. Nine products (30.0%) were from *Pu. cernua*, implying that *Pu. cernua* holds a considerable share as a substitute plant source of Baitouweng on the Chinese market. *Pu. cernua* is mainly located in Heilongjiang, Jilin, Liaoning, and Nei Mongol within China, but also occurs in Japan, Korea, and Russia. Although it was not stipulated in Chinese Pharmacopoeia, it was noted as plant origin of medicinal Baitouweng in “Chinese Herb Medicine”^32^. Thus, these congeneric substitutes may have similar medicinal efficacy with Baitouweng, and merit further testing. The second type of adulterants that we detected in medicinal market samples were contaminants from unrelated species. Baitouweng is an inexpensive, but easily confused herb. Plants with white hairs near the root are always misidentified as the origin plant. Therefore, contaminants are mainly due to misidentification, and not through intentional adulteration. Our finding indicated that 16 Baitouweng products (53.3%) belonged to this type, including *Po. chinensis, R. uniflorum, A. hupehensis*, and *P. grandiflorus.* Among them, *Potentilla* species might be the most common contaminant of medicinal Baitouweng in the market. *Po. chinensis* is also a commonly used TCM with Chinese vernacular name Weilingcai^22^ and is often be reported as adulterant of Baitouweng^33^,^35^. Additionally, plants from the *Gossypium* and *Astragalus* were also detected in this study. Therefore, ITS2 barcoding can effectively authenticate commercial Baitouweng products and its adulterants, aiding in solving the security issues of medicinal Baitouweng in the market.

DNA barcoding is becoming a helpful method for authenticating bioingredients in commercial herbal product. Newmaster *et al*. reported contamination and substitution in North American herbal products using a tiered DNA barcoding approach (*rbcL* + ITS2)^44^. Bruni *et al*. used the *rbcL* and *psbA-trnH* regions to identify the taxonomic composition of honey in multiflower honey, and detected one toxic plant in one sample^45^. Stoeckle *et al*. also identified some unlisted ingredients in herbal teas^46^. Xin *et al*. distinguished super food *Lycium barbarum* from its closely related species via ITS2 barcoding^47^. For authenticating commercial *Pu. chinensis* and its adulterants, choosing an ideal DNA barcode is very important. As our result, the ITS2 region is a suitable one for this goal. Firstly, the ITS2 barcode is much shorter than other candidate barcodes and can easily be amplified even for some degraded DNA samples^15^. The manufacturing process often degrades the crude form of Baitouweng. In this study, the ITS2 region was 100% amplified and successfully sequenced for all fresh samples and commercial products. Secondly, the ITS2 barcode has a high identification power^48^,^49^, and has already identified many medicinal materials successfully^47^,^50^,^51^,^52^. Our study indicated that using the ITS2 barcode could accurately authenticate genuine medicinal Baitouweng of *Pu. chinensis* from its adulterants. A similar result was reported that this region enabled a molecular discrimination of *Pu. patens* and *Pu. vernalis*^53^. Therefore, ITS2 barcoding is an effective tool for authenticating commercial Baitouweng products from its adulterants. But for mixed herbal products, obtaining ITS2 sequences by TA cloning is still laborious and time-consuming. Next-Generation Sequencing (NGS) represents a new vision to detect the organic ingredients in mixed samples, such as tablets, powders, capsules and pills^54^,^55^,^56^, and will provide an efficient and cost-effective mean to detect species composition of mixed TCM products. Targeting both DNA and characteristic components by DNA sequencing and chromatographic analysis will be a better integrative approach for authenticating TCM products, especially for standardized extracts with degraded DNA^57^.

### Quick identification of *Pu. chinensis* via specific PCR-RFLP assay based on the ITS2 region

**Figure 5:**
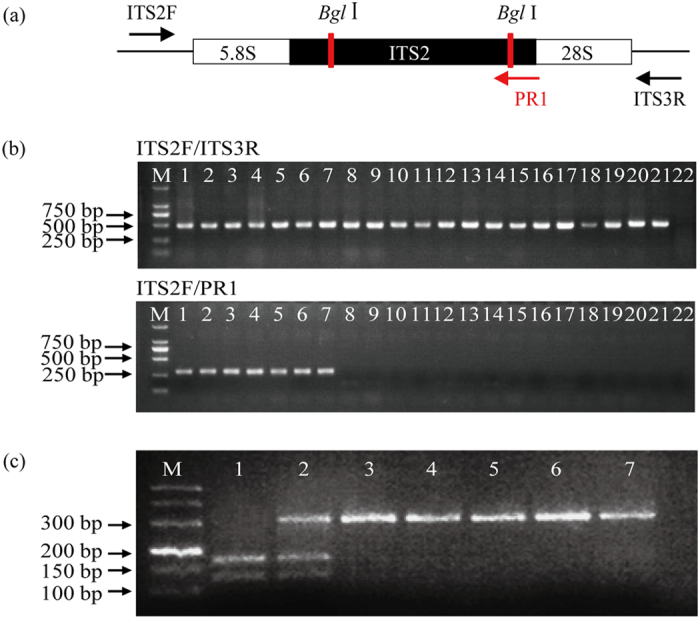
Specific PCR-RFLP assay of *Pulsatilla chinensis* and its adulterants. (**a**) Specific primers for *Pulsatilla* species and *Bgl* I sites within the ITS2 region of *Pu. chinensis*. (**b**) PCR products by primers ITS2F/ITS3R and ITS2/PR1. Lane 1–22: *Pu. chinensis* (1–2), *Pu. cernua* (3–4), *Pu. campanella* (5–6), *Pu. millefolium* (7), *Anemone hupehensis* (8–9), *Cimicifuga simplex* (10–11), *Clematis armandii* (12–13), *Potentilla chinensis* (14–15), *Po. discolor* (16–17), *Rhaponticum uniflorum* (18–19), *Platycodon grandiflorus* (20–21) and water control (22). Lane M, DL2000 DNA marker. (**c**) *Bgl* I restriction digest pattern of PCR products of *Pulsatilla* species. Lane 1–7: *Pu. chinensis* (1–2), *Pu. cernua* (3–4), *Pu. campanella* (5–6) and *Pu. millefolium* (7). Lane M, DL500 DNA marker. Full-length gels are presented in Supplementary Figure S3.

A distinctive restriction site of *Bgl* I (GCCNNNNNGGC) in the ITS2 region of *Pu. chinensis* was selected by SNP analysis (Fig. 5a); thus, a PCR-RFLP assay was developed for quick identification of *Pu. chinensis*. We designed a specific PCR primer pair ITS2F/PR1 and replaced the thirteenth G in PR1 with T to avoid another *Bgl* I site in *Pulsatilla* species (Fig. 5a), thus PCR amplicons by ITS2/PR1 will have no *Bgl* I site in *Pulsatilla* species except *Pu. chinensis*. Then, four *Pulsatilla* species and two closely related species (*Cimicifuga simplex* Wormsk and *Clematis armandii* Franch) and other four adulterant species of Baitouweng were selected to test this assay. PCR results indicated that the DNA were of good quality for PCR amplification because the ITS2 regions were all successfully amplified by ITS2F/ITS3R. Moreover, ITS2/PR1 were able to successfully amplify approximately 292 bp products from four *Pulsatilla* species but no PCR product were present in other species (Fig. 5b), indicating ITS2/PR1 is highly specific for *Pulsatilla* species and can significantly discriminate them from other species. *Bgl* I digestion results showed that only two PCR amplicons from *Pu. chinensis* were cleaved into two expected fragments of approximately 168 bp and 124 bp lengths, others could not be digested any more (Fig. 5c). Although one PCR product of *Pu. chinensis* was not digested completely, that might be due to the small PCR fragment. However, this did not affect the identification result. Therefore, using the specific PCR-RFLP primers ITS2/PR1 combined with restriction enzyme *Bgl* I can not only identify *Pulsatilla* species from others, but also authenticate *Pu. chinensis* from congeneric species. In addition, 30 commercial Baitouweng products were also examined by the specific PCR-RFLP assay. The identification results were consistent with ITS2 barcoding results discussed previously (Table 3), indicating that this assay was suitable for market samples.

In conclusion, we presented two molecular approaches for identifying medicinal *Pu. chinensis* from its adulterants. ITS2 barcoding can accurately identify *Pu. chinensis* and its adulterants by DNA sequencing, thereby providing the sequence basis for species identification. The specific PCR-RFLP assay based on the ITS2 region can rapidly identify authenticate the genuine plant origin *Pu. chinensis* of Baitouweng from others without DNA sequencing. Although specific PCR-RFLP assay cannot identify the certain species of adulterants, it offers a faster, simpler, and less expensive manner for identifying commercial Baitouweng products and more convenient for market survey.

## Methods

### Materials and taxon sampling

A total of 84 voucher samples derived from 13 species were collected from the provinces of Henan, Shaanxi, Hubei, Jilin, Beijing, Guangxi, Shandong, Chongqing, Anhui and Yunnan in China. These include: *Pu. chinensis, Pu. cernua, Pu. campanella, Pu. millefolium, Po. chinensis, Po. discolor, R. uniflorum, P. grandiflorus, A. hupehensis, A. decumbens, L. leontopodioides, D. cappa*, and *G. affine* (see Supplementary Table S1). All corresponding voucher samples are deposited in the herbarium of the Institute of Medicinal Plant Development, Chinese Academy of Medicinal Sciences (IMD). 21 sequences were downloaded from GenBank, the accession numbers being JN811072, JN811073, JN811074, JN811075, HQ829820, JN811070, JN811071, JF422892, JF422893, JF422894, JF422890, HQ735289, GU732648, FJ639908, JF422891, GU732651, GU732649, HQ440207, FJ639944, GU126788, and KP092567. Additionally, 30 medicinal Baitouweng products were purchased from different drug stores and markets in China, and one sample from each product was randomly selected to test.

### ITS2 barcoding: DNA extraction, PCR amplification, sequencing and data analysis

All fresh samples and incomplete dried commercial products were dried with silica gel. 30 mg of each sample was powdered for 2 min at 50 Hz in a tissue grinder (Sceintz-48, China). Total genomic DNA was isolated using the Plant Genomic DNA Kit (Tiangen Biotech Co., China). The ITS2 region was amplified using the published universal primers ITS2F (5′-ATGCGATACTTGGTGTGAAT-3′) and ITS3R (5′-GACGCTTCTCCAGACTACAAT-3′)^15^, 25 μL PCR reaction volume containing <100 ng of genomic DNA, 2X Taq PCR MasterMix (Aidlab Biotechnologies Co., China), 1 μL of each primer (2.5 μM, synthesized by Sangon Co., China), and distilled-deionized water. PCR conditions were 1 cycle of 95 °C for 3 min, 35 cycles of 94 °C for 30 s, 56 °C for 30 s, and 72 °C for 40 s followed by 1 cycle of 72 °C for 10 min. PCR products (6 μL of each) were examined on 1.2% agarose gel electrophoresis in 1 × TAE buffer for 20 minutes at 120 V. Purified PCR products were sequenced bidirectionally with PCR primers on an ABI-3730 sequencer (Applied Biosystem, USA). For TA cloning, purified PCR products were cloned into pMD18T vector, transformed into *E. coli* DH5αcompetent cells, and then screened positive monoclonies and sequenced as previously described.

Sequences were assembled using Codoncode aligner v. 4.2.4. The complete ITS2 sequences were annotated based on the Hidden Markov Model (HMM)^58^. All ITS2 sequences were deposited in GenBank and DNA barcoding system for identifying herbal medicine (http://www.tcmbarcode.cn/en/). Then, seven nucleotides at the 3′terminus were cut off after sequence alignment to keep all sequences consistent. Sequence alignment was performed by ClustalW in MEGA5.1. The intra- and inter-specific distances were calculated using the K2P model^59^ and the phylogenetic tree was constructed using the NJ method in MEGA5.1^60^. DNA barcoding database for herbal materials (http://www.tcmbarcode.cn/en/) and nucleotide database in National Center for Biotechnology Information (NCBI) (http://blast.ncbi.nlm.nih.gov/Blast.cgi) were used as sources for complementary ITS2 data for species identification.

### Specific PCR-RFLP assay

PCR amplification was carried out with ITS2F and PR1 (5′-TGGGTCATCTTCTCCCAGC-3′ in a final volume of 25 μL as described before. The PCR conditions were 1 cycle of 95 °C for 3 min, 35 cycles of 94 °C for 30 se, 62 °C for 20 s, and 72 °C for 20 s followed by 1 cycle of 72 °C for 10 min. PCR products (6 μL of each) were visualized by 2% agarose gel electrophoresis in 1 × TAE buffer for 20 minutes at 120 V.

Restriction enzymes were predicted by Vector NTI 10.3.0 (Invitrogen, Carlsbad, CA, USA) and *Bgl* I was selected as the best. Digestion was performed by incubating 15 μL of PCR product with 10 U of *Bgl* I (NEB) and 2.5 μL 10 × buffer in a final volume of 25 μL at 37 °C for 1 h, and then restriction fragments were separated by 3% agarose gel electrophoresis in 1 × TAE buffer for 30 min at 120 V.

## Additional Information

**How to cite this article:** Shi, Y. *et al*. Rapidly discriminate commercial medicinal *Pulsatilla chinensis* (Bge.) Regel from its adulterants using ITS2 barcoding and specific PCR-RFLP assay. *Sci. Rep.* **7**, 40000; doi: 10.1038/srep40000 (2017).

**Publisher's note:** Springer Nature remains neutral with regard to jurisdictional claims in published maps and institutional affiliations.

## Supplementary Material

Supplementary Information

## Figures and Tables

**Table 1:** Characteristics of the ITS2 sequences in the reference database.

Specie	Sample number	Sequence length (bp)	Average GC content (%)	Variable sites
*Pulsatilla chinensis*	25	211	64.5	4
*Pu. cernua*	9	212	64.0	1
*Pu. campanella*	8	212	64.0	9
*Pu. millefolium*	1	212	—	—
*Pu. tongkanensis*	4	212	64.6	0
*Pu. violacea*	3	11	64.6	3
*Pu. dahurica*	2	212	64.0	8
*Pu. albana*	1	211	64.9	—
*Pu. halleri*	1	210	65.2	—
*Pu. rubra*	1	212	65.6	—
*Pu. sp.*	1	212	65.2	—
*Pu. turczaninovii*	1	212	64.2	—
*Potentilla chinensis*	10	204–206	64.3	20
*Po. discolor*	8	204	63.2	3
*Rhaponticum uniflorum*	10	216	57.5	4
*Platycodon grandiflorus*	6	255	63.9	0
*Anemone hupehensis*	4	204–205	67.0	2
*Ajuga decumbens*	4	221	63.9	1
*Leontopodium leontopodioides*	2	216	51.6	0
*Duhaldea cappa*	2	223	51.0	0
*Gerbera piloselloides*	1	222	59.0	—
*Gnaphalium affine*	1	217	49.8	—
All species	105	204–255	61.9	—

**Table 2:** The intra- and inter-specific K2P distance of ITS2 sequences.

Species	KP2 distances of the ITS2		
	Minimum	Maximum	Mean
*Pulsatilla chinensis* & *Pu. chinensis*	0	0.019	0.006
*Pu. chinensis* & other 11 *Pulsatilla* species	0.005	0.060	0.020
*Pu. chinensis* & adulterants of other genera	0.113	0.755	0.595

**Table 3:** Authentication of commercial Baitouweng products.

Sample codes	Products forms	ITS2 barcoding			specific PCR	PCR-RFLP
		Sequence length (bp)	Blast identified organism	Maximum identity (%)		
Y1	root	211	*Pulsatilla chinensis*	100	+	+
Y2	root	212	*Pu. campanella*	99.5	+	−
Y3	root	212	*Pu. cernua*	100	+	−
Y4	root	212	*Pu. cernua*	100	+	−
Y5	root	211	*Pu. chinensis*	99.5	+	+
Y6	root	212	*Pu. dahurica*	99.5	+	−
Y7	root	212	*Pu. cernua*	100	+	−
Y8	root	212	*Pu. cernua*	100	+	−
Y9	root	212	*Pu. cernua*	100	+	−
Y10	root	212	*Pu. cernua*	100	+	−
Y11	root	212	*Pu. dahurica*	99.5	+	−
Y12	root and leaf	204	*Potentilla chinensis*	100	−	N/A
Y13	root and leaf	204	*Po. chinensis*	100	−	N/A
Y14	root and leaf	204	*Po. chinensis/ Po. nivea*	98.1	−	N/A
Y15	root and leaf	204	*Po. chinensis*	100	−	N/A
Y16	root and leaf	204	*Po. chinensis*	100	−	N/A
Y17	root	204	*Po. chinensis*	99.0	−	N/A
Y18	root	204	*Po. chinensis/ Po. discolor*	100	−	N/A
Y19	root and leaf	204	*Po. chinensis/ Po. discolor*	100	−	N/A
Y20	root	216	*Rhaponticum uniflorum*	100	−	N/A
Y21	root	216	*R. uniflorum*	100	−	N/A
Y22	root	216	*R. uniflorum*	100	−	N/A
Y23	root	204	*Anemone hupehensis*	100	−	N/A
Y24	root	205	*A.hupehensis*	100	−	N/A
Y25	root	255	*Platycodon grandiflorus*	100	−	N/A
Y26	root	212	*Pu. cernua*	100	+	−
Y27	root	212	*Pu. cernua*	100	+	−
Y28	root	212	*Pu. cernua*	100	+	−
Y29	root	224	*Gossypium hirsutum/ G. barbadense*	100	−	N/A
Y30	powder	216	*R. uniflorum*	98.2–100	−	N/A
		210	*Atragalus* genus	99.1–99.5		
