# Supplementary material for: Rapidly discriminate commercial medicinal Pulsatilla chinensis (Bge.) Regel from its adulterants using ITS2 barcoding and specific PCR-RFLP assay
Source: Sci Rep. 2017 Jan 6;7:40000. doi: 10.1038/srep40000 (PMC5216359; doi:10.1038/srep40000)
Supplement: Supplementary Information [file srep40000-s1.pdf]

Rapidly discriminate commercial medicinal *Pulsatilla chinensis* (Bge.) Regel from its adulterants using ITS2 barcoding and specific PCR-RFLP assay

Yuhua Shi, Mingming Zhao, Hui Yao, Pei Yang, Tianyi Xin, Bin Li, Wei Sun*, Shilin Chen*

Supplementary Information

Supplementary Table S1 Information of voucher samples used in the study

Species	Family	Sample ID	Voucher number	Collected part	Collected place	GenBank accession
Pulsatilla chinensis	Ranunculaceae	B1	PS0899B1	leaf	Nanyang, Henan	KR611721
Pu. chinensis	Ranunculaceae	B2	PS0899B2	leaf	Nanyang, Henan	KR611722
Pu. chinensis	Ranunculaceae	B3	PS0899B3	leaf	Nanyang, Henan	KR611723
Pu. chinensis	Ranunculaceae	B4	PS0899B4	leaf	Nanyang, Henan	KR611724
Pu. chinensis	Ranunculaceae	B5	PS0899B5	leaf	Nanyang, Henan	KR611725
Pu. chinensis	Ranunculaceae	B6	PS0899B6	leaf	Nanyang, Henan	KR611726
Pu. chinensis	Ranunculaceae	B7	PS0899B7	root	Nanyang, Henan	KR611727
Pu. chinensis	Ranunculaceae	B8	PS0899B8	root	Nanyang, Henan	KR611728
Pu. chinensis	Ranunculaceae	B9	PS0899B9	root	Nanyang, Henan	KR611729
Pu. chinensis	Ranunculaceae	B10	PS0899B10	root	Nanyang, Henan	KR611730
Pu. chinensis	Ranunculaceae	B11	PS0899B11	leaf	Xi'an, Shaanxi	KR611731
Pu. chinensis	Ranunculaceae	B12	PS0899B12	leaf	Xi'an, Shaanxi	KR611732
Pu. chinensis	Ranunculaceae	B13	PS0899B13	leaf	Xi'an, Shaanxi	KR611733
Pu. chinensis	Ranunculaceae	B14	PS0899B14	leaf	Shengnongjia, Hubei	KR611734
Pu. chinensis	Ranunculaceae	B15	PS0899B15	leaf	Shengnongjia, Hubei	KR611735
Pu. chinensis	Ranunculaceae	B16	PS0899B16	leaf	Shengnongjia, Hubei	KR611736
Pu. chinensis	Ranunculaceae	B17	PS0899B17	leaf	Baihuashan, Beijing	KR611737
Pu. chinensis	Ranunculaceae	B18	PS0899B18	leaf	Baihuashan, Beijing	KR611738
Pu. chinensis	Ranunculaceae	B19	PS0899B19	leaf	Baihuashan, Beijing	KR611739
Pu. chinensis	Ranunculaceae	B20	PS0899B20	leaf	Changbaishan, Jilin	KR611740
Pu. chinensis	Ranunculaceae	B21	PS0899B21	leaf	Changbaishan, Jilin	KR611741
Pu. chinensis	Ranunculaceae	B22	PS0899B22	leaf	Changbaishan, Jilin	KR611742
Pu. chinensis	Ranunculaceae	B23	PS0899B23	leaf	Beijing	KR611743
Pu. chinensis	Ranunculaceae	B24	PS0899B24	leaf	Beijing	KR611744
Pu. chinensis	Ranunculaceae	B25	PS0899B25	leaf	Beijing	KR611745
Pu. cernua	Ranunculaceae	C1	PS3102C1	leaf	Dandong, Liaoning	KT969417
Pu. cernua	Ranunculaceae	C2	PS3102C2	leaf	Dandong, Liaoning	KT969418
Pu. cernua	Ranunculaceae	C3	PS3102C3	leaf	Dandong, Liaoning	KT969419
Pu. cernua	Ranunculaceae	C4	PS3102C4	leaf	Dandong, Liaoning	KT969420
Pu. cernua	Ranunculaceae	C5	PS3102C5	leaf	Dandong, Liaoning	KT969421
Pu. cernua	Ranunculaceae	C6	PS3102C6	leaf	Dandong, Liaoning	KT969422
Pu. cernua	Ranunculaceae	—	—	—	GenBank	HQ829820
Pu. cernua	Ranunculaceae	—	—	—	GenBank	JN811070
Pu. cernua	Ranunculaceae	—	—	—	GenBank	JN811071
Pu. campanella	Ranunculaceae	Z1	PS3103Z1	leaf	Wulumuqi, Xinjiang	KT969423
Pu. campanella	Ranunculaceae	Z2	PS3103Z2	leaf	Wulumuqi, Xinjiang	KT969424
Pu. campanella	Ranunculaceae	Z3	PS3103Z3	leaf	Wulumuqi, Xinjiang	KT969425
Pu. campanella	Ranunculaceae	Z4	PS3103Z4	leaf	Wulumuqi, Xinjiang	KT969426

Pu. campanella	Ranunculaceae	Z5	PS3103Z5	leaf	Wulumuqi, Xinjiang	KT969427
Pu. campanella	Ranunculaceae	Z6	PS3103Z6	leaf	Wulumuqi, Xinjiang	KT969428
Pu. campanella	Ranunculaceae	Z7	PS3103Z7	leaf	Wulumuqi, Xinjiang	KT969429
Pu. campanella	Ranunculaceae	Z8	PS3103Z8	leaf	Wulumuqi, Xinjiang	KT969430
Pu. millefolium	Ranunculaceae	X1	PS3104X1	leaf	Yunnan	KT969431
Pu. tongkanensis	Ranunculaceae	—	—	—	GenBank	JN811072
Pu. tongkanensis	Ranunculaceae	—	—	—	GenBank	JN811073
Pu. tongkanensis	Ranunculaceae	—	—	—	GenBank	JN811074
Pu. tongkanensis	Ranunculaceae	—	—	—	GenBank	JN811075
Pu. violacea	Ranunculaceae	—	—	—	GenBank	JF422892
Pu. violacea	Ranunculaceae	—	—	—	GenBank	JF422893
Pu. violacea	Ranunculaceae	—	—	—	GenBank	JF422894
Pu. albana	Ranunculaceae	—	—	—	GenBank	JF422890
Pu. dahurica	Ranunculaceae	—	—	—	GenBank	HQ735289
Pu. dahurica	Ranunculaceae	—	—	—	GenBank	GU732648
Pu. halleri	Ranunculaceae	—	—	—	GenBank	FJ639908
Pu. rubra	Ranunculaceae	—	—	—	GenBank	JF422891
Pu. sp.	Ranunculaceae	—	—	—	GenBank	GU732651
Pu. turczaninovii	Ranunculaceae	—	—	—	GenBank	GU732649
Potentilla chinensis	Rosaceae	W1	PS1120W1	leaf	Shengnongjia, Hubei	KR611746
Po. chinensis	Rosaceae	W2	PS1120W2	leaf	Shengnongjia, Hubei	KR611747
Po. chinensis	Rosaceae	W3	PS1120W3	leaf	Shengnongjia, Hubei	KR611748
Po. chinensis	Rosaceae	W4	PS1120W4	leaf	Chongqing	KR611749
Po. chinensis	Rosaceae	W5	PS1120W5	leaf	Chongqing	KR611750
Po. chinensis	Rosaceae	W6	PS1120W6	leaf	Wuhan, Hubei	KR611751
Po. chinensis	Rosaceae	W7	PS1120W7	leaf	Wuhan, Hubei	KR611752
Po. chinensis	Rosaceae	W8	PS1120W8	leaf	Wuhan, Hubei	KR611753
Po. chinensis	Rosaceae	W9	PS1120W9	leaf	Beijing	KR611754
Po. chinensis	Rosaceae	W10	PS1120W10	leaf	Beijing	KR611755
Po. discolor	Rosaceae	F1	PS1079F1	leaf	Beijing	KR611766
Po. discolor	Rosaceae	F2	PS1079F2	leaf	Beijing	KR611767
Po. discolor	Rosaceae	F3	PS1079F3	leaf	Nanning, Guangxi	KR611768
Po. discolor	Rosaceae	F4	PS1079F4	leaf	Nanning, Guangxi	KR611769
Po. discolor	Rosaceae	F5	PS1079F5	leaf	Nanning, Guangxi	KR611770
Po. discolor	Rosaceae	F6	PS1079F6	leaf	Nanning, Guangxi	KR611771
Po. discolor	Rosaceae	F7	PS1079F7	leaf	Nanning, Guangxi	KR611772
Po. discolor	Rosaceae	F8	PS1079F8	leaf	Anhui	KR611773
Rhaponticum uniflorum	Compositae	L1	PS0674L1	leaf	Nanyang, Henan	KR611756
R. uniflorum	Compositae	L2	PS0674L2	root	Beijing	KR611757
R. uniflorum	Compositae	L3	PS0674L3	root	Beijing	KR611758
R. uniflorum	Compositae	L4	PS0674L4	leaf	Beijing	KR611759
R. uniflorum	Compositae	L5	PS0674L5	leaf	Songshan, Henan	KR611760
R. uniflorum	Compositae	L6	PS0674L6	leaf	Songshan, Henan	KR611761
R. uniflorum	Compositae	L7	PS0674L7	leaf	Songshan, Henan	KR611762

R. uniflorum	Compositae	L8	PS0674L8	leaf	Changbaishan, Jilin	KR611763
R. uniflorum	Compositae	L9	PS0674L9	leaf	Changbaishan, Jilin	KR611764
R. uniflorum	Compositae	L10	PS0674L10	leaf	Changbaishan, Jilin	KR611765
Platycodon grandiflorus	Campanulaceae	J1	PS0583J1	leaf	Qingdao, Shandong	KR611774
P. grandiflorus	Campanulaceae	J2	PS0583J2	leaf	Qingdao, Shandong	KR611775
P. grandiflorus	Campanulaceae	J3	PS0583J3	leaf	Chongqing	KR611776
P. grandiflorus	Campanulaceae	J4	PS0583J4	leaf	Chongqing	KR611777
P. grandiflorus	Campanulaceae	J5	PS0583J5	leaf	Chongqing	KR611778
P. grandiflorus	Campanulaceae	J6	PS0583J6	leaf	Chongqing	KR611779
Ajuga decumbens	Labiatae	JC1	PS1738JC1	leaf	Anhui	KR611783
A. decumbens	Labiatae	JC2	PS1738JC2	leaf	Anhui	KR611784
A. decumbens	Labiatae	JC3	PS1738JC3	leaf	Chongqing	KR611785
A. decumbens	Labiatae	JC4	PS1738JC4	leaf	Yunnan	KR611786
Anemone hupehensis	Ranunculaceae	D1	PS3105D1	leaf	Shengnongjia, Hubei	KR611780
A.hupehensis	Ranunculaceae	D2	PS3105D2	leaf	Shengnongjia, Hubei	KR611781
A.hupehensis	Ranunculaceae	D3	PS3105D3	leaf	Shengnongjia, Hubei	KR611782
A.hupehensis	Ranunculaceae		—	—	GenBank	HQ440207
Leontopodium leontopodioides	Compositae		PS0634MT01	leaf	Donglingshan, Beijing	FJ980329
L. leontopodioides	Compositae		—	—	GenBank	FJ639944
Duhaldea cappa	Compositae		PS0701MT01	leaf	Kunming, Yunnan	FJ980352
D. cappa	Compositae		—	—	GenBank	KP092567
Gerbera piloselloides	Compositae		—	—	GenBank	GU126788
Gnaphalium affine	Compositae		PS0684MT01	leaf	Putian, Fujian	FJ980346

Supplementary Table S2 Intraspecific distances of species in the ITS2 database of *Pulsatilla chinensis* and its adulterants

Specie	Sample number	Intraspecific ITS2 distances (KP2)		
		Minimum	Maximum	Mean
Pulsatilla chinensis	25	0	0.019	0.006
Pu. cernua	9	0	0.005	0.002
Pu. campanella	9	0	0.029	0.012
Pu. millefolium	1	—	—	—
Pu. tongkanensis	4	0	0	0
Pu. violacea	3	0.005	0.014	0.010
Pu. dahurica	2	0.029	0.029	0.029
Pu. albana	1	—	—	—
Pu. halleri	1	—	—	—
Pu. rubra	1	—	—	—
Pu. sp.	1	—	—	—
Pu. turczaninovii	1	—	—	—
Potentilla chinensis	10	0	0.078	0.046
Po. discolor	8	0	0.015	0.005
Rhaponticum uniflorum	10	0	0.014	0.007
Platycodon grandiflorus	6	0	0	0
Anemone hupehensis	4	0	0.010	0.004
Ajuga decumbens	4	0	0.005	0.002
Leontopodium leontopodioides	2	0	0	0
Duhaldea cappa	2	0	0	0
Gerbera piloselloides	1	—	—	—
Gnaphalium affine	1	—	—	—

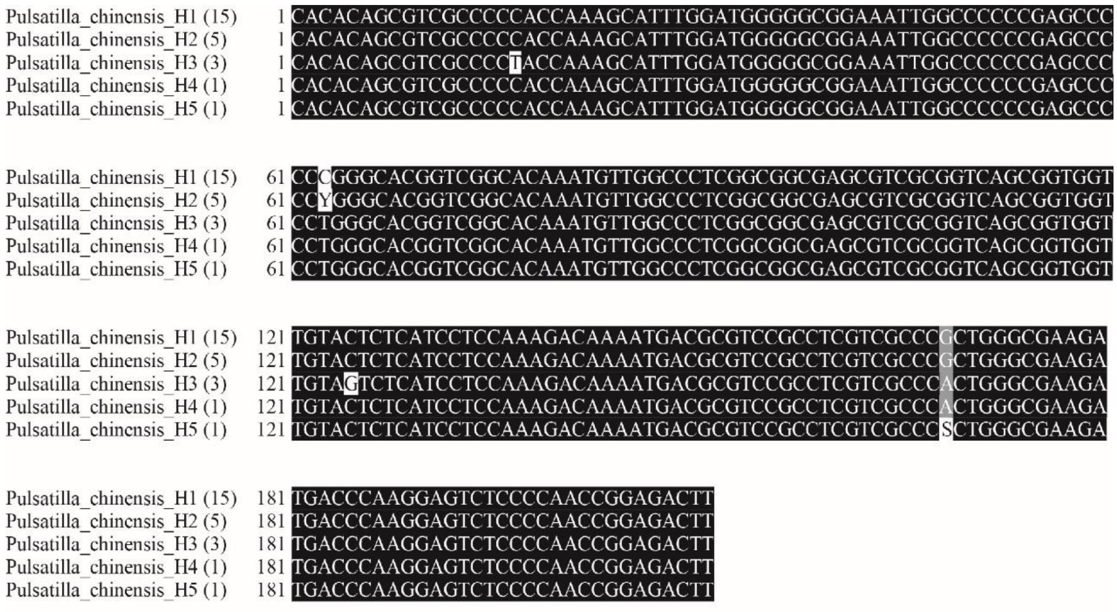

Supplementary Figure S1 Intragenomic variation in the ITS2 sequences of *Pulsatilla chinensis*. The figure was performed by ClustalW-BOXSHADE alignment method. Each digit in the bracket represents the number of the ITS2 haplotype; black shade indicates identical base; gray or white shade indicates variable base; Y represents T/C; S represents C/G.

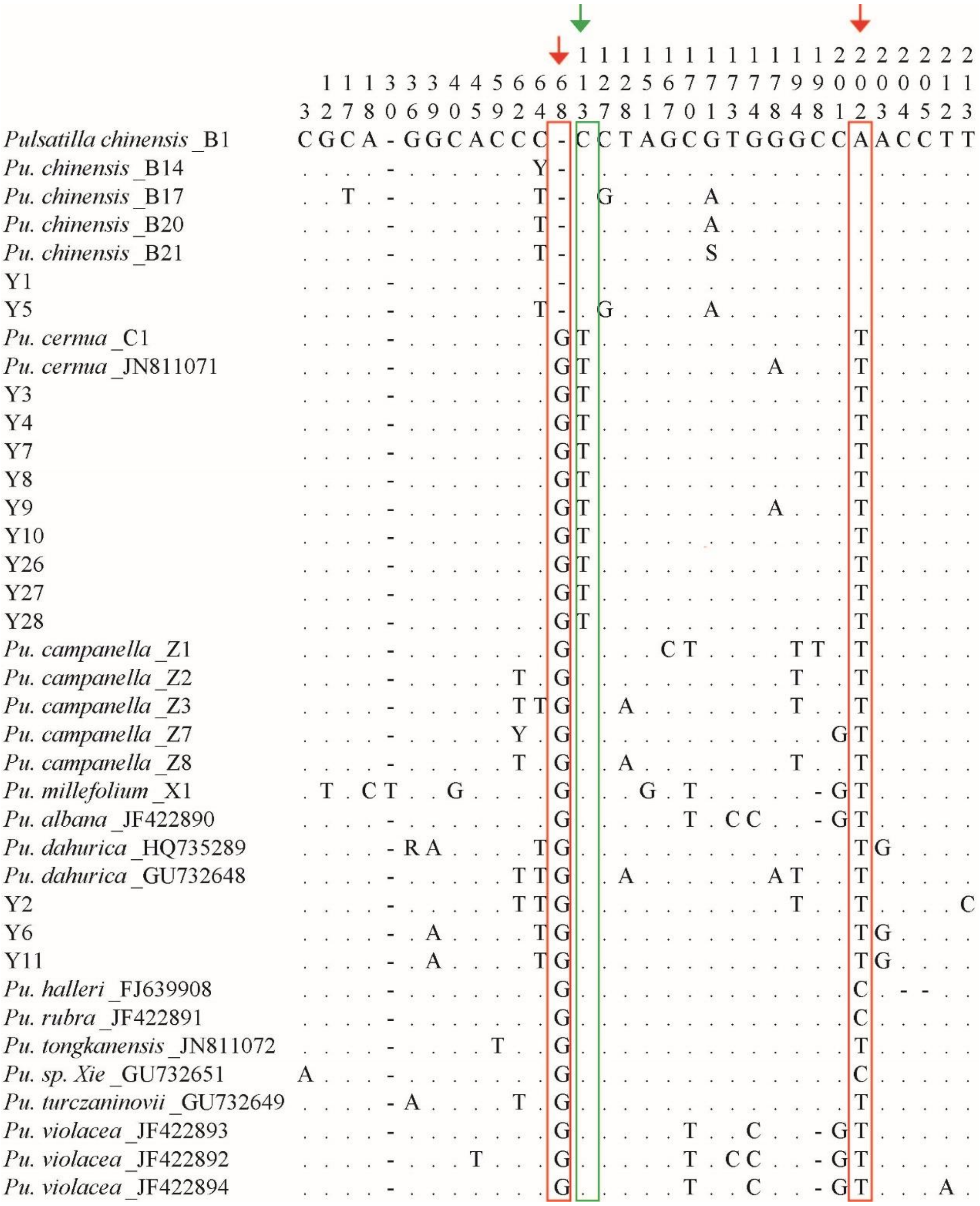

Supplementary Figure S2 Sequence divergence among *Pulsatilla* species and fourteen commercial Baitouweng products in the ITS2 region**.** The number represents the positions of variable sites (bp); dots (.) represents the nucleotide identical to the first line; dash (–) represents a nucleotide indel; Y represents T/C; S represents C/G; R represents A/G. Red arrows and boxes indicate the specific SNP sites in *Pu. chinensis*; green arrow and box indicate the specific SNP sites in *Pu. crenua.*

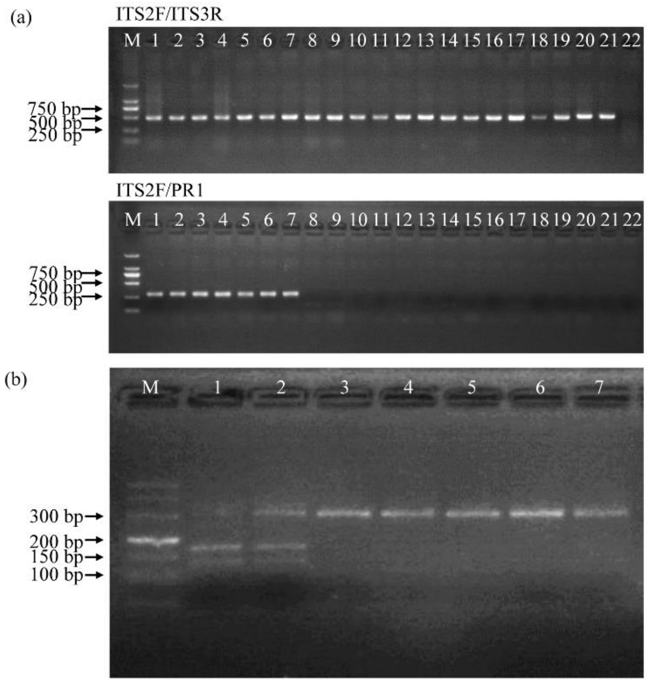

Supplementary Figure S3 Full-length gels of Specific PCR-RFLP assay of *Pulsatilla chinensis* and its adulterants. (a) PCR products by primers ITS2F/ITS3R and ITS2/PR1. Lane 1-22: *Pu. chinensis* (1-2), *Pu. cernua* (3-4), *Pu. campanella* (5-6), *Pu. millefolium* (7), *Anemone hupehensis* (8-9), *Cimicifuga simplex* (10-11), *Clematis armandii* (12-13), *Potentilla chinensis* (14-15), *Po. discolor* (16-17), *Rhaponticum uniflorum* (18-19), *Platycodon grandiflorus* (20-21) and water control (22). Lane M, DL2000 DNA marker. (b) *Bgl* I restriction digest pattern of PCR products of *Pulsatilla* species. Lane 1-7: *Pu. chinensis* (1-2), *Pu. cernua* (3-4), *Pu. campanella* (5-6) and *Pu. millefolium* (7). Lane M, DL500 DNA marker.
